# Kynurenines in CNS disease: regulation by inflammatory cytokines

**DOI:** 10.3389/fnins.2014.00012

**Published:** 2014-02-06

**Authors:** Brian M. Campbell, Erik Charych, Anna W. Lee, Thomas Möller

**Affiliations:** Neuroinflammation Disease Biology Unit, Lundbeck Research USAParamus, NJ, USA

**Keywords:** kynurenine, neuroinflammation, microglia, astrocytes, CNS disease, IDO, KMO, KAT

## Abstract

The kynurenine pathway (KP) metabolizes the essential amino acid tryptophan and generates a number of neuroactive metabolites collectively called the kynurenines. Segregated into at least two distinct branches, often termed the “neurotoxic” and “neuroprotective” arms of the KP, they are regulated by the two enzymes kynurenine 3-monooxygenase and kynurenine aminotransferase, respectively. Interestingly, several enzymes in the pathway are under tight control of inflammatory mediators. Recent years have seen a tremendous increase in our understanding of neuroinflammation in CNS disease. This review will focus on the regulation of the KP by inflammatory mediators as it pertains to neurodegenerative and psychiatric disorders.

## The kynurenine pathway

**Figure 1:**
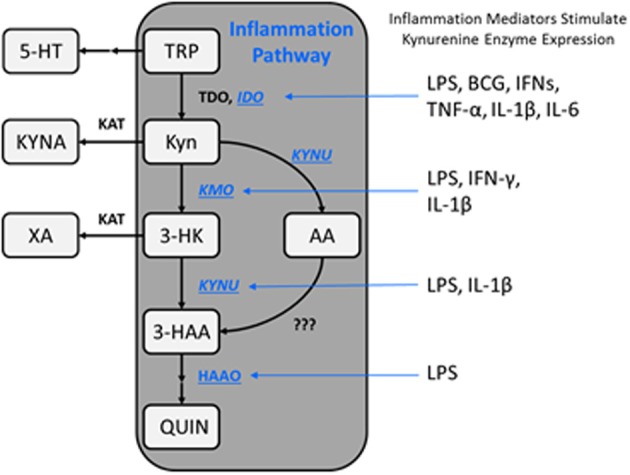
**Schematic representation of the kynurenine metabolic pathway**. The kynurenine pathway is commonly segregated into two distinct branches that are regulated by KATs and KMO, as well as the availability of l-kynurenine within the brain. Additionally, kynurenine metabolism is regulated by a variety of proinflammatory mediators which impact enzyme expression levels, thereby altering substrate availability and metabolite formation favoring the KMO branch of the pathway under immune-related pathological conditions. TRP, tryptophan; 5-HT, serotonin; Kyn, kynurenine; KYNA, kynurenine acid; 3-HK, 3-hydroxykynurenine; AA, anthranilic acid; XA, xanthurenic acid; 3-HAA, 3-hydroxyanthranilic acid; QUIN, quinolinic acid; IDO, indoleamine-2,3-dioxygenase; KAT, kynurenine aminotransferase; KMO, kynurenine 3-monooxygenase; KYNU, kynureninase; HAAO, 3-hydroxyanthranilic acid oxidase; LPS, lipopolysaccharide; BCG, bacillus Calmette-Guerin; IFNs, interferons; TNF, tumor necrosis factor; IL, interleukin.

The metabolic fate of tryptophan (TRP), an essential amino acid, is conversion into a variety of neuroactive substances including the well-known neurotransmitters serotonin and melatonin, as well as a range of kynurenine metabolites such as kynurenic acid (KYNA), 3-hydroxykynurenine (3-HK), and quinolinic acid (QUIN). Enzymes involved in the metabolism of tryptophan along the kynurenine pathway (KP) are located thoughout the body and brain. Though the highest levels are found in the liver and kidney, all of the primary enzymes are also found within the brain. Kynurenine metabolism occurs in all cells within the brain, though various branches of the pathway appear segregated into specific cell types (Heyes et al., 1997; Amori et al., 2009). The first and rate-limiting enzyme into the KP is indole-2,3-dioxygenase (IDO), and to a lesser extent in the brain tryptophan-2,3-dioxygenase (TDO), which convert tryptophan to N-formylkynurenine (Shimizu et al., 1978; Takikawa et al., 1988) (for a schematic of the pathway see Figure 1). N-formylkynurenine is then metabolized to l-kynurenine (L-KYN) by kynurenine formamidase at which point the pathway bifurcates into at least two distinct branches regulated by kynurenine monooxygenase (KMO) and kynurenine aminotransferases (KATs I-IV). The majority of kynurenine metabolism within the brain takes place in glia. KMO, kynureninase (KYNU), and 3-hydroxyanthranillic acid oxidase (3-HAO) regulate production of a host of metabolites in microglia leading to formation of anthranillic acid (AA), 3-hydroxy anthranillic acid (3-HAA), 3-HK, and QUIN. QUIN is, an excitatory (excitotoxic) agent at NMDA-type glutamate receptors and synergizes with 3-HK to produce oxidative stress. Alternatively, L-KYN may be metabolized in astrocytes by KATs, with KAT II being the predominant brain subtype in humans and rats (Guidetti et al., 2007a). KATs convert L-KYN to KYNA, an inhibitor of glutamate neurotransmission and possibly an antagonist at nicotinic α_7_ receptors. The endogenous function of kynurenine-derived neuroactive metabolites still requires further research since many have multiple receptor targets. In addition to NMDA and nicotinic a7 receptors, KYNA for example is reported to interact with GPR35 (Wang et al., 2006) and arylhydrocarbon receptors (Dinatale et al., 2010). A third possible pathway regulated by both KMO and KATs is the xanthurenic acid (XA) branch. Little is known about the endogenous function of XA, though recent literature indicates that it is a Group II metabotropic glutamate receptor agonist (Copeland et al., 2013) indicating that it could also regulate glutamate neurotransmission by impacting presynaptic release.

In recent years the regulation of kynurenine metabolism has been intensely evaluated as it relates to CNS disorders (Haroon et al., 2012; Schwarcz et al., 2012). Often termed the “neurotoxic” and “neuroprotective” branches of the KP, or alternatively the “excitatory” and “inhibitory” branches, KMO and KATs regulate the balance of QUIN:KYNA production which is important in both neurodegenerative and psychiatric disorders. Many kynurenine-derived metabolites poorly cross the blood brain barrier implying that CNS concentrations of kynurenine metabolites are largely regulated by local enzyme activity (Gal and Sherman, 1978). However, kynurenine itself is actively transported into the brain by the large neutral amino acid transporter (Fukui et al., 1991). Under normal physiological conditions much of the kynurenine which is converted to QUIN and KYNA in the brain is derived from peripheral sources (Kita et al., 2002). Following systemic inflammation, where IDO expression is greatly increased (Moreau et al., 2008; Macchiarulo et al., 2009), nearly all kynurenine in the CNS comes from the periphery. However, in contrast to this, direct induction of neuroinflammation causes >98% of the kynurenine available for metabolism in the brain to be derived from local production (Kita et al., 2002). The current review will evaluate this interplay between proinflammatory mediators and mechanisms by which they regulate the KP. It will then conclude with a review of the role of neuroinflammation-mediated kynurenine dysregulation in a range of neurodegenerative and psychiatric disorders.

## Cytokine-mediated regulation of kynurenine metabolism

IDO and TDO, which initiate the catabolism of tryptophan toward kynurenine, are generally thought to be regulated by different mechanisms. TDO is induced by corticosteroids and glucagon, while IDO is induced by proinflammatory cytokines during an immune response (Lestage et al., 2002). There is some evidence that TDO can also be induced by immune activation but this is suggested to be mediated indirectly by increased glucocorticoid receptor activation (Walker et al., 2013). While there is some evidence that other enzymes within the excitatory branch of the KP can also be induced by proinflammatory cytokines, the regulation of IDO, particularly by interferon (IFN)-γ, has been examined most extensively. In general, the body of work investigating the regulation of KP enzymes by inflammatory cytokine signaling is largely composed of expression studies and therefore must be interpreted with caution, since changes in mRNA or even protein expression are not necessarily indicative of functional changes in enzyme activity.

### Effects of proinflammatory mediators on indoleamine 2,3-dioxygenase (IDO)

IDO is expressed in various immune cells throughout the body, including dendritic cells, monocytes, macrophages, and, importantly in microglia, the resident CNS macrophage-like cell population (Mandi and Vecsei, 2012). IDO is preferentially induced by interferons and by IFN-inducers such as lipopolysaccharide (LPS) and viruses (Musso et al., 1994). IFN-γ, a type II interferon, is the predominant cytokine implicated in the induction of IDO, as has been shown in several myeloid cell types including dendritic cells, monocytes, immortalized murine macrophages, and microglia (Alberati-Giani et al., 1996; Fujigaki et al., 2006; Jung et al., 2007; O'connor et al., 2009a). In human macrophages, IDO expression and QUIN production can also be induced by the type 1 interferons, IFN-α and IFN-β, although to a lesser degree than with IFN-γ (Jansen and Reinhard, 1999; Guillemin et al., 2001). In the bacille Calmette-Guérin (BCG) mouse model of chronic inflammation, IDO induction closely parallels increased IFN-γ and tumor necrosis factor alpha (TNF-α) expression (Moreau et al., 2005, 2008). BCG-induced upregulation of IDO mRNA is completely inhibited in IFN-γR^−/−^ mice, along with an associated lack of IDO activity, demonstrating that IFN-γ receptor function is necessary for BCG-induced IDO activation (O'connor et al., 2009a).

Although IFN-γ is regarded as the primary inducer of IDO, there is some evidence that IDO expression can be induced independently of IFN-γ. Systemic LPS administration induces IDO expression in rat cortex and hippocampus accompanied by a robust increase in central TNF-α and interleukin (IL)-6 expression, but only modestly elevated IFN-γ (Connor et al., 2008). In the same paper, similar findings were reported in mixed glia cultures prepared from neonatal rat cortex suggesting that IFN-γ may not be necessary for LPS-induced IDO expression (Connor et al., 2008). Consistent with this finding, *in vitro* data with THP-1 cells, a human monocytic cell line, indicate that LPS-induced IDO activation can be mediated by an IFN-γ-independent mechanism involving synergistic effects of IL-1β, TNF-α, and IL-6 (Fujigaki et al., 2006). In human hippocampal progenitor cells, treatment with IL-1β greatly upregulated the transcript for IDO, but not TDO (Zunszain et al., 2012). The increase in IDO transcript was associated with a decrease in tryptophan and increase in kynurenine in the supernatant suggesting that IL-1β increased levels of functional IDO enzyme (Zunszain et al., 2012).

Studies examining the effects of anti-inflammatory cytokines on IDO expression are limited and often conflicting, likely due to differences in the cellular models used and experimental conditions applied. For example, the prototypical anti-inflammatory cytokine IL-10 dose-dependently decreased LPS-mediated IDO protein expression in mouse bone marrow-derived dendritic cells (BMDCs), whereas IL-10 enhanced IFN-γ-mediated IDO protein expression in these cells (Jung et al., 2009; Yanagawa et al., 2009). This discrepancy may point to the possibility that distinct mechanisms of IDO induction may be differentially regulated by anti-inflammatory cytokines such as IL-10, though whether this occurs in the CNS has not been determined. Interestingly, however, IL-10 suppressed IFN-γ-mediated IDO mRNA induction in GT1-7 cells, a transformed mouse hypothalamic neuronal cell line, contrary to that reported for mouse BMDCs treated with IFN-γ (Tu et al., 2005). In addition to the prototypical anti-inflammatory cytokine IL-10, studies with human monocytes and fibroblasts have demonstrated that IL-4 inhibits the induction of IDO mRNA and IDO activity by IFN-γ. In contrast, a study using the EOC13.31 mouse microglia cell line found that IL-4 enhanced, rather than suppressed, IFN-γ-induced IDO mRNA expression, which was abolished by the addition of IL-4 antiserum (Yadav et al., 2007). The potentiating effect of IL-4 on IFN-γ-induced IDO expression was also observed at the level of protein expression and enzymatic activity in these cells (Yadav et al., 2007). Furthermore, IL-4, as well as IL-13 which signals through the same receptor subunit, potentiated IFN-γ-mediated IDO expression in primary mouse microglia cultures (Yadav et al., 2007). These findings collectively suggest that microglia respond differently to anti-inflammatory cytokines compared to peripheral myeloid cells. Interestingly, central administration of IL-4 exacerbates the depressive-like behavioral effect of peripheral LPS, which is IDO-dependent, when both IL-4 and LPS are delivered simultaneously, but suppresses the depressive effect when administered 12 h before LPS, highlighting the complex relationship between IL-4 and IDO in the CNS (Bluthe et al., 2002).

#### IFN-γ-dependent mechanisms of IDO induction

**Figure 2:**
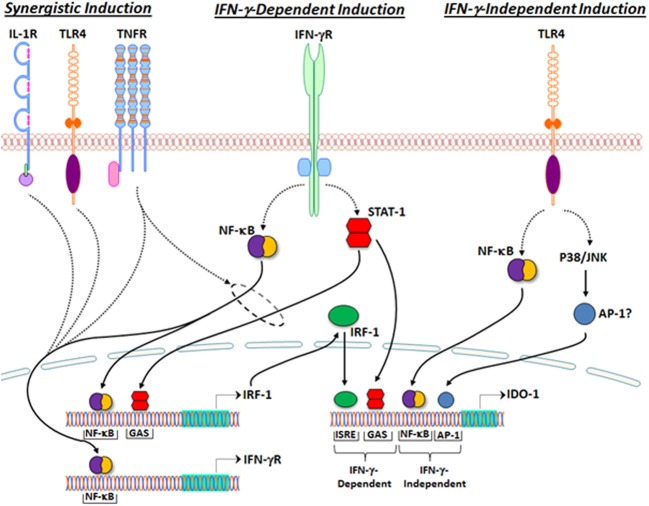
**Regulation of IDO1 transcription by inflammatory signaling**. IFN-γ-dependent IDO1 induction (**middle**). Canonical IFN-γ receptor signal transduction leads to (1) NF-κB- and STAT-1-dependent transcription of IRF-1, and (2) IRF-1- and STAT-1-dependent transcription of IDO1. Synergistic IDO1 induction (**Left**). IL-1β, LPS, and TNF-α enhance transcription of IFN-γ receptor in an NF-κB-dependent manner. TNF-α has been shown to synergistically enhance IFN-γ-dependent IDO1 transcription by promoting NF-κB- and STAT-1-dependent IRF-1 transcription (within dashed circle). IFN-γ-Independent IDO induction (**Right**). TLR4 stimulation by LPS leads to transcription of IDO1 by a mechanism that requires NF-κB and either p38 or JNK, but not IFN-γ. The 5′-flanking region of *INDO*, the gene encoding IDO1, contains two IFN-γ-activated sites (GAS) and two interferon-sensitive response elements (ISREs). One of the two GAS sequences and both ISRE sequences are required for IFN-γ-mediated IDO1 induction. The 5′ flanking region of *INDO* also contains at least one NF-κB binding site and several AP-1 binding sites, which may be required for IFN-γ-independent mechanisms of IDO1 transcription.

The 5′-flanking region of the human gene encoding IDO (*INDO)* contains several regulatory elements including some that are essential for IFN-γ-mediated gene transcription. One of two identified IFN-γ-activated sites (GAS) and two interferon-sensitive response elements (ISREs), the latter highly homologous to that associated with IFN-α-inducible genes, are required for full induction of IDO by IFN-γ (Dai and Gupta, 1990; Hassanain et al., 1993; Chon et al., 1995, 1996; Konan and Taylor, 1996). As shown in Figure 2, canonical IFN-γ-mediated signal transduction leads to (1) tyrosine phosphorylation of STAT-1, triggering its dimerization and translocation to the nucleus where it binds the GAS sequence in the 5′-flanking region of *INDO*, and (2) NF-κB- and STAT-1-dependent synthesis of IFN-γ-regulated factor (IRF)-1, which binds to one or both of the ISREs found in the *INDO* 5′-flanking region (Darnell et al., 1994; Chon et al., 1995, 1996; Konan and Taylor, 1996). Thus, cooperative STAT-1 and IRF-1 binding to GAS and ISRE sequences, respectively, within the *INDO* 5′-flanking region are necessary for full IFN-γ-mediated induction of IDO transcription.

#### Synergistic mechanisms of IFN-γ-mediated IDO induction

The regulatory mechanisms for IFN-γ-mediated IDO induction can be potentiated by other proinflammatory cytokines such as TNF-α and IL-1β, and toll-like receptor (TLR) agonists such as LPS, resulting in synergistic enhancement of IDO expression (Hu et al., 1995; Hissong and Carlin, 1997; Babcock and Carlin, 2000; Currier et al., 2000; Robinson et al., 2003). IL-1β and TNF-α can enhance the expression of IFN-γ receptor in an NF-κB-dependent manner, thereby lowering the threshold for IDO induction by IFN-γ (Krakauer and Oppenheim, 1993; Shirey et al., 2006). Moreover, together with IFN-γ, TNF-α synergistically induces IDO expression by increasing both STAT-1 activation and NF-κB-dependent IRF-1 expression (Krakauer and Oppenheim, 1993; Ohmori et al., 1997; Robinson et al., 2003, 2006; Shirey et al., 2006). Given the requirement for both STAT-1 and IRF-1 binding to ISRE and GAS sequences, respectively, presumably other signaling mechanisms that increase both STAT-1 phosphorylation and NF-κB transactivation may also synergize with IFN-γ to enhance IDO induction, though these mechanisms have not yet been directly tested. Interestingly, the synergistic induction of IDO by IFN-γ and TNF-α occurs in primary murine microglia and, furthermore, *in vivo* studies suggest that this synergy participates in the IDO-mediated generation of depressive-like behavior in mice inoculated with BCG (O'connor et al., 2009a), a model of inflammation-related depression (Moreau et al., 2008).

#### IFN-γ-independent mechanisms of IDO induction

Studies using primary murine microglia demonstrated that LPS stimulates IDO transcription in an IFN-γ-independent manner, since IDO mRNA levels were enhanced but IFN-γ mRNA was undetectable following LPS stimulation in these cells (Connor et al., 2008; Wang et al., 2010). Furthermore, these studies showed that LPS-stimulated IDO induction was attenuated by an inhibitor of c-Jun-N-terminal kinase (JNK) (Wang et al., 2010). Similar studies using THP-1 cells, demonstrated that LPS-stimulated L-KYN production was not accompanied by STAT-1 or IRF-1 binding activities, but was attenuated by p38 and NF-κB inhibitors (Fujigaki et al., 2001, 2006). Collectively, these data suggest that LPS-stimulated IDO induction in monocyte/macrophage-like cells occurs in an IFN-γ-independent manner and involves NF-κB and stress-activated mitogen-activated protein (MAP) kinases such as p38 and JNK (Fujigaki et al., 2001, 2006, 2012; Wang et al., 2010). The downstream mechanisms leading from p38 or JNK activation to IDO induction in response to LPS stimulation have not been elucidated. However, the AP-1 transcription factors are conventional substrates of both p38 and JNK MAPKs and are important regulators of inflammation-related gene transcription (Huang et al., 2009; Wang et al., 2010). Supporting this possibility, a reanalysis of the 5′-flanking region of *INDO* has identified both NF-κB and several AP-1 recognition sequences, consistent with the participation of both NF-κB and stress-activated MAPK activity in LPS-stimulated IDO induction (Fujigaki et al., 2006; Wang et al., 2010).

In addition to TLR4 agonists such as LPS, the TLR3 agonist polyinosinic:polycytidylic acid (polyI:C) can induce IDO transcription in cultured human astrocytes in a manner dependent on IFN-β but not IFN-γ signaling, and requiring both NF-κB and IRF-3 (Suh et al., 2007). Though these signaling components have been shown to participate in astrocyte IDO induction, it is not yet clear whether the corresponding mechanism can be generalized to cell types other than astrocytes since the effect of TLR3 activation on IDO induction has not been demonstrated elsewhere.

#### Aryl hydrocarbon receptor-dependent IDO induction

Experiments using murine BMDCs have demonstrated that the TLR4 and TLR9 agonists LPS and CpG, respectively, induce expression of the aryl hydrocarbon receptor (AhR). The AhR is a ligand-gated transcription factor belonging to the basic helix-loop-helix Per-Arnt-Sim (PAS) family, widely known as the dioxin receptor (Vogel et al., 2008; Nguyen et al., 2010; Vondracek et al., 2011). Interestingly, these experiments suggested that LPS- or CpG-stimulated IDO induction was entirely dependent on the co-induction of AhR in these cells, since BMDCs derived from AhR^−/−^ mice lost the ability to induce IDO expression in response to treatment with either LPS or CpG (Nguyen et al., 2010). Furthermore, dioxin, a potent agonist of the AhR, can also induce IDO expression in these cells, suggesting that AhR activation may positively regulate IDO transcription in response to TLR4 or TLR9 stimulation (Nguyen et al., 2010). Intriguingly, AhR-mediated IDO induction may act as a positive feedback mechanism further activating AhR since L-KYN and its metabolite KYNA are themselves potent AhR agonists (Dinatale et al., 2010; Opitz et al., 2011). The AhR exerts its effects on gene transcription through nuclear translocation and direct binding to dioxin response elements (DREs) in the promoter region of target genes. Curiously these elements have not been identified in the promotor region of *INDO*. Thus, it is not clear whether AhR can regulate IDO transcription directly or indirectly in these cells.

### Effects of proinflammatory mediators on kynurenine-3-monooxygenase (KMO)

Aside from IDO, the regulation of other kynurenine enzymes by proinflammatory cytokines has not been studied extensively. However, studies are emerging indicating that, similar to IDO, enzymes within the KMO branch of the pathway may also be induced by proinflammatory stimuli. KMO expression is increased in rat brain after systemic LPS administration (Connor et al., 2008; Molteni et al., 2013). In a study that examined the effects of IFN-γ treatment on immortalized murine macrophage (MT2) and microglia (N11) cells, KMO was induced in both cells types, KYNU was induced only in MT2 macrophages, and 3-HAO was not effected (Alberati-Giani et al., 1996). Finally, in human hippocampal progenitor cells, IL-1β treatment upregulated the level of transcripts for KMO and KYNU, enzymes in the KMO branch of the pathway (Zunszain et al., 2012).

### Effects of proinflammatory mediators on kynurenine aminotransferases (KATs)

While the expression of IDO and kynurenine enzymes in the excitatory branch of the KP are either elevated or not changed by proinflammatory stimuli, KAT expression is either unaffected or decreased. Systemic LPS administration had no effect on KAT II expression in rat brain (Connor et al., 2008; Molteni et al., 2013). In MT2 macrophage and N11 microglia cells, KAT appeared to be constitutively expressed, but there was no effect of IFN-γ treatment on KAT activity (Alberati-Giani et al., 1996). However, since in the CNS KATs are mainly expressed in astrocytes, further studies on the effects of proinflammatory stimuli on KAT expression and activity using relevant cell types are required. In human hippocampal progenitor cells, KAT I and KAT III, but not KAT II mRNA, were downregulated after IL-1β treatment (Zunszain et al., 2012).

## Dysregulation of the kynurenine pathway in CNS diseases

In recent years dysregulation of kynurenine metabolism has been described in a wide range of CNS-related disorders. Several studies have demonstrated that altered cytokine levels and associated dysregulation of kynurenine metabolism plays as important role in the pathophysiology of neurodegenerative diseases and psychiatric disorders. Upregulation of kynurenines are observed in the serum, CSF and/or brain in neurodegenerative diseases (e.g., AD, PD, and HD), autoimmune diseases (e.g., MS), epilepsy, psychiatric diseases (e.g., MDD, schizophrenia, and ADHD) and infectious diseases (e.g., HIV-associated neurocognitive disorder). It is generally predicted that diseases where microglia are activated favor production of 3-HK and QUIN, whereas suppression of this branch or astrocyte activation may favor KYNA synthesis. The following sections will review the role of the kynurenine system and its regulation by cytokines in the pathophysiology of diseases, and discuss potential therapeutic interventions targeting the KP.

### Alzheimer's disease

Alzheimer's disease (AD) is a progressive neurological disorder characterized by impaired memory, cognitive decline, and dementia. Currently there is still only a limited understanding of AD etiology, particularly in late onset AD. AD pathology hallmarks are the presence of β-amyloid (Aβ) plaques, neurofibrillary tangles, and gliosis. Multiple hypotheses exist regarding factors that contribute to the development and progression of AD including substantial evidence for neuroinflammatory processes. In fact, microglia activation states correlate with disease progression and levels of dementia (Arends et al., 2000; Cagnin et al., 2006). Analysis of serum samples and post-mortem brain tissue from AD patients demonstrate an imbalance in pro- and anti-inflammatory cytokines, as well as irregular tryptophan metabolism through activation of microglia and astrocytes.

#### (Neuro)inflammatory state in AD

Among the neurochemical changes in AD, IFN-γ, TNF-α, IL-1β, IL-2, and IL-8 are elevated along with lower levels of tryptophan and increased kynurenine levels in serum samples from AD patients (Widner et al., 1999; Alsadany et al., 2013; Niranjan, 2013). Similar changes are found in post-mortem brain tissue along with IL-6 also increased (Huell et al., 1995). Within the brains of AD patients, activated microglia and astrocytes are found in proximity to neuritic plaques. Treatment of human microglia and monocytes with Aβ_1−42_ induces IDO expression (Guillemin et al., 2003) and primes the cells for synergistic induction of the KP by IFN-γ (Yamada et al., 2009). In astrocytes Aβ only modestly stimulated IL-6 and IL-8 secretion, but primed the cells to markedly respond to IL-1β with a 3–8 fold increase in IL-6 and IL-8 release (Gitter et al., 1995). Similarly, exposure of microglia cultures from AD patients to Aβ_1−42_ induced TNF-α, pro-IL-1β, IL-6, and IL-8 (Lue et al., 2001). Thus, Aβ appears to alter the state of microglia to a more proinflammatory phenotype that may contribute to neuronal dysfunction and ultimately cell death through release of cytokines and free radical generating agents including NO and QUIN. In AD brains IDO was associated with senile plaques and was localized with neurofibrillary tangles (Bonda et al., 2010). Additionally, IDO and QUIN immunoreactivity were increased in microglia, astrocytes, and neurons within the hippocampus of AD patients (Guillemin et al., 2005) which is of particular interest since QUIN may cause tau hyperphosphorylation in human cortical neurons (Rahman et al., 2009).

#### Inflammation and kynurenine metabolism in animal models of AD

Studies in preclinical models support the hypothesis that induction of kynurenine metabolism by Aβ and/or cytokines may contribute to neural pathology in AD. Elevated Aβ_1−40_ and Aβ_1−42_ found in transgenic AD mice were associated with increased TNF-α, IL-6, and IL-1β (Patel et al., 2005). In Tg2576 mice, basal induction of IDO in activated microglia associated with Aβ plaques appears to be low, though robustly increased following stimulation with LPS suggesting that the cells are in a “primed” state ready to respond to immune challenges in a more durable way than WT controls (Akimoto et al., 2007). QUIN was strongly increased in the hippocampus, but not cerebellum, in a progressive and age dependent manner in triple transgenic mice (3 × Tg: PS1M146V, APPSwe, and tauP301L) in line with data showing increased TDO and IDO-1 immunoreactivity in AD hippocampal tissue (Wu et al., 2013). Interestingly, modest but significant increases in TDO mRNA and protein, along with robust increases in 3-HAO were also found in the cerebellum of these mice, however, 3-HAA levels were unfortunately not reported. Furthermore, TDO was also colocalized with QUIN, neurofibrillary tangles, and amyloid deposits in 3 × Tg mice. Recent studies using available pharmacological agents in AAPtg mice showed that chronic inhibition of KMO reduced synaptic loss as measured by synaptophysin, prevented spatial memory deficits in the Morris water maze, and reversed anxiogenic-like responses in the elevated plus maze (Zwilling et al., 2011). Together these data support the hypothesis that Aβ - and cytokine-mediated induction of kynurenine metabolism is an important link in the pathophysiological development of AD.

Induction of kynurenine metabolism, particularly along the KMO/QUIN branch of the pathway, appears likely in AD. The mechanism by which this happens, and the functional consequences, is still under investigation. To date, much of the data available is correlative indicating that Aβ is able to induce production of cytokines and kynurenine metabolizing enzymes which may both contribute to synaptic dysfunction and neuronal loss. Whether Aβ in AD brains independently induces cytokine production and kynurenine metabolism, activates cytokine release which in turn stimulates kynurenine production, or “primes” glia such that they are able to more robustly respond to cytokine signaling is currently not well-understood. Whichever the case, evidence is emerging that excess production of proinflammatory cytokines and QUIN by glia contribute to the progression, and perhaps etiology, of AD.

Preclinical evidence supports the use of IDO, TDO, KMO, and/or 3-HAO inhibitors to counteract the effects of neuroinflammation in AD. However, the contribution of peripheral vs. central inflammatory processes in any putative kynurenine-related AD pathology is not yet clear, and few of the tools available to test these hypotheses are able to directly target the brain. Association of IDO and TDO with plaques and tangles suggests that brain permeable drugs are needed, though peripheral inhibition of KMO was sufficient to produce a therapeutic effect in Tg2576 mice. Since the source of kynurenine feeding into the QUIN branch differs substantially under basal, systemic inflammation, or neuroinflammation conditions, understanding the contribution of central vs. peripheral (including endothelial cells at the blood brain barrier) kynurenine production will be important to help define an appropriate intervention strategy in AD.

### Parkinson's disease

Parkinson's disease (PD) is a chronic progressive neurodegenerative disorder characterized by loss of dopaminergic neurons in the midbrain and presence of protein inclusions called Lewy bodies (Zinger et al., 2011). The detailed pathogenesis of PD is not known, but several mechanisms have been proposed including mitochondrial dysfunction, neurotoxicity from excessive glutamatergic activity, and reactive oxygen species. Neuroinflammation, as measured by the presence of activated microglia in PD brain, as well as excessive production of cytokines and dysregulation of the KP have been suggested to be involved in these complex pathogenic events.

#### (Neuro)inflammatory state in PD

Many studies support the presence of widespread microglia activation in PD. In two such studies, MHC class II expression, a widely used marker of microglial activation, was assessed in PD post-mortem brain (McGeer et al., 1988; Imamura et al., 2003). The number of MHC class II-positive microglia was higher in the substantia nigra and putamen as well as in the hippocampus, transentorhinal cortex, cingulate cortex, and temporal cortex of PD brains, and frequently in association with α-synuclein-positive Lewy neurites and monoaminergic neurites (McGeer et al., 1988; Imamura et al., 2003). These activated microglia were also positive for TNF-α and IL-6 in the putamen of PD brain (Imamura et al., 2003). *In vivo* imaging of microglia activation with [^11^C](R)-PK11195 PET in PD revealed widespread activation in brain regions including the pons, basal ganglia, and frontal and temporal cortex (Gerhard et al., 2006). Levels of several cytokines including TNF-α, IL-1β, IL-2, IL-4, IL-6, and transforming growth factor (TGF)-alpha have been shown to be elevated in the CSF and striatum of PD brain (Mogi et al., 1994a,b; Nagatsu et al., 2000). Some of these cytokines are known inducers or amplifiers of the KP and might contribute to the dysregulation of KPs in PD.

#### Dysregulation of kynurenine metabolites in PD

Changes in kynurenine metabolism have been reported in post-mortem PD brain and mouse models of PD. In mouse models of PD, mice injected with the dopaminergic neurotoxins 1-methyl-4-phenyl-1,2,3,6-tetrahydropyridine (MPTP) or 6-hydroxydopamine have diminished KAT-I immunoreactivity in the pars compacta of the substania nigra (Knyihar-Csillik et al., 2004, 2006). Treatment with the metabolite of MPTP, 1-methyl-4-phenylpyridinium ion (MPP^+^), dose-dependently decreased KAT-II activity and KYNA concentration in rat cerebral cortical slices (Luchowski et al., 2002). Similar to the KYNA changes observed in rodent models of PD, KYNA levels were reported to be decreased in PD post-mortem brain (Ogawa et al., 1992). In contrast, levels of 3-HK were elevated in the frontal cortex, putamen, and pars compacta of the substantia nigra in PD brain (Ogawa et al., 1992). In terms of IDO activity, as measured by the K/T ratio, there were increases in both serum and CSF of PD patients compared to controls (Widner et al., 2002). Taken together, these studies suggest that there is an imbalance between the two main branches of the KP in PD, favoring kynurenine metabolism toward the KMO branch of the pathway.

#### Potential therapeutic intervention by modulation of kynurenine pathway

Numerous studies have been conducted demonstrating that modulation of the KP by enhancing KYNA and/or decreasing 3-HK and QUIN is a potential therapeutic strategy for PD. In an *in vitro* PD model, pretreatment with KYNA attenuated MPP^+^-induced neurotoxicity in human neuroblastoma cell lines (Lee Do et al., 2008). In rats, KYNA injection into the brain prevented QUIN-induced reduction in striatal tyrosine hydroxylase activity, suggesting that KYNA can protect dopaminergic neurons against QUIN or NMDA-mediated excitotoxicity (Miranda et al., 1997). Since KYNA does not cross the blood brain barrier, investigators in one study attempted to increase KYNA levels in the brain with systemic injections of the substrate for KYNA, L-KYN, in combination with probenecid, an inhibitor of organic acid transport (Silva-Adaya et al., 2011). They reported that pretreatment with L-KYN and probenecid had a protective effect on 6-OHDA-induced locomotor asymmetry, striatal reactive gliosis and neurodegeneration, and changes in dopamine levels (Silva-Adaya et al., 2011). In another study, four synthetic kynurenine analogs were demonstrated to have beneficial effects in the MPTP model in mice (Acuna-Castroviejo et al., 2011). Finally, in MPTP-treated primates, intracerebral injections of KYNA alleviated symptoms of akinesia, tremor, and rigidity in MPTP-treated animals (Graham et al., 1990). Thus, KYNA or its analogs have been demonstrated to have neuroprotective effects in PD.

The effect of decreasing metabolites in the KMO branch of the KP and thereby increasing KYNA in the brain has been tested pharmacologically by several investigators. When nicotinylalanine, an inhibitor of both KMO and kynureninase, was administered intraventricularly in combination with systemic L-KYN and probenecid, there was an elevation in brain KYNA levels and protective effects against QUIN-induced toxicity in rats (Miranda et al., 1997). The effect of KMO inhibition has been tested in a variety of animal models of PD with the non-brain penetrant KMO inhibitor Ro 61-6048. Ro 61-6048 given either systemically or intrastriatally, reduced the severity of dystonia in *dt^sz^*mutant hamsters, which are used as an animal model of paroxysmal dystonia with striatal dysfunctions (Richter and Hamann, 2003; Hamann et al., 2008). In non-human primates, chronic Ro 61-8048 administration reduced the development of levodopa-induced dyskinesia in MPTP-treated animals (Samadi et al., 2005; Gregoire et al., 2008; Ouattara et al., 2009; Tamim et al., 2010). Taken together, these studies indicate that drug development targeting the KP for PD may be a promising opportunity.

### Huntington's disease

Huntington's disease (HD) is an adult-onset neurodegenerative disorder caused by expansion of a CAG repeat in the gene encoding the huntingtin (Htt) protein. Several mechanisms that are not mutually exclusive have been suggested to play a role in the pathogenesis of HD. These mechanisms include neuroinflammation, transcriptional dysregulation, excitotoxicity, and mitochondrial dysfunction. Damage of mutant Htt expressing neurons is suggested to lead to microglia activation, which includes secretion of cytokines as well as increased IDO transcription and generation of neuroactive kynurenine metabolites (Schwarcz and Pellicciari, 2002). Indeed, increased levels of metabolites have been reported in human post-mortem brain as well as in various animal models of HD as discussed below.

#### (Neuro)inflammatory state in HD

Several lines of evidence suggest both peripheral and central immune systems are activated in HD. Activation of the peripheral immune system is indicated by elevation in several plasma cytokines in HD patients including IL-6, IL-8, IL-4, IL-10, and TNF-α (Bjorkqvist et al., 2008). Interestingly, plasma levels of IL-6 were elevated on average 16 years prior to the predicted clinical onset of the disease in HD gene carriers, which is the earliest plasma abnormality identified in HD (Bjorkqvist et al., 2008). In a study that examined blood levels of kynurenine metabolites at different stages of HD, levels of IL-2 were found to be correlated with the K/T ratio, disease severity, and number of CAG repeats (Forrest et al., 2010).

Activation of the immune system in the CNS is evidenced by elevations in IL-6 and IL-8 in the CSF and increased expression of these inflammatory transcripts, as well as by increased TNF-α in post-mortem HD brain (Bjorkqvist et al., 2008; Silvestroni et al., 2009). There is ample evidence that microglia, the main mediators of neuroinflammation, contribute to the progressive neurodegeneration observed in HD (Möller, 2010). Interestingly they are also the main producers of 3-HK and QUIN in the CNS. Given the presence of IDO and KMO inducing enzymes and the data showing increased KP metabolism in HD and HD model brains, it is tempting to speculate that an increased flux through the microglial KMO metabolic pathway might be responsible for these observations.

#### Dysregulation of kynurenine metabolites in HD

Studies examining post-mortem HD brain found elevations in the levels of 3-HK and QUIN (Pearson and Reynolds, 1992; Guidetti et al., 2000, 2004). The activity of 3-HAO, the biosynthetic enzyme in the metabolism of 3-HAA, was increased in HD brains compared to controls, suggesting that the HD brain has the ability to produce elevated levels of QUIN (Schwarcz et al., 1988). On the other hand, levels of KYNA and the activity of its two biosynthetic enzymes (KAT I and KAT II) were reported to be reduced in HD brain and CSF compared to controls (Beal et al., 1990, 1992; Jauch et al., 1995) suggesting a dysregulation of the KP in the brain away from KYNA and toward QUIN.

R6/2 mice, a well-established model of HD, also have elevated 3-HK in the brain and have increased activity of the biosynthetic enzyme of 3-HK, KMO, which may account for the high levels (Guidetti et al., 2006; Sathyasaikumar et al., 2010). YAC128 transgenic mice, which have the full-length mutant Htt protein and show a similar degree of striatal neurodegeneration observed in early stage HD, have elevated 3-HK and QUIN in the brain (Guidetti et al., 2000, 2006). Intriguingly, QUIN injections into the striatum is commonly used as an experimental model of HD and produces cellular, neurochemical and behavioral changes resembling those observed in human HD (Beal et al., 1991; Huang et al., 1995).

Dysregulation of the KP, as measured by the K/T ratio, a marker of IDO activity, has been reported in the periphery as well (Stoy et al., 2005; Forrest et al., 2010). One study examined levels of kynurenine metabolites in the blood of patients at different stages of HD as well as the number of CAG repeats and found blood levels of K/T ratio were correlated with disease severity and the number of CAG trinucleotide repeats in HD patients (Forrest et al., 2010). In the same study, blood levels of anthranilic acid were correlated with the proinflammatory cytokine IL-23 (Forrest et al., 2010). Taken together, these studies suggest a role of dysregulation of the KP in HD which may be related to the degree of clinical disease severity.

#### Potential therapeutic intervention by modulation of kynurenine pathway in huntington's disease

Studies in yeast, flies, and mice, have shown that blockade of the KMO branch of the KP, thus increasing KYNA in the brain, may protect against neurodegeneration. Genetic deletion of KMO in yeast cells engineered to over express mutated huntingtin protein reduced polyglutamine-mediated toxicity as well as generation of the neuroactive kynurenine metabolites 3HK and QUIN (Giorgini et al., 2005). Furthermore, when a high throughput screen was conducted on the yeast model an analog of the KMO inhibitor 3,4-dimethoxy-N-[4-(3-nitrophenyl)thiazol-2-yl]benzenesulfonamide (Ro61-8048) was identified that potently suppressed huntingtin-mediated toxicity (Giorgini et al., 2005). In transgenic *Drosophila melanogaster* flies that express mutant Htt protein, genetic or pharmacological blockade of KMO reduced neuronal cell loss (Campesan et al., 2011). In the R6/2 genetic mouse model of HD, peripheral blockade of KMO increased KYNA in the brain, reduced loss of synapses and microglia activation, and improved survival (Zwilling et al., 2011). In N171-82Q mice, another transgenic animal model of HD, a KYNA analog, N-(2-N,N-dimethylaminoethyl)-4-oxo-1H-quinoline2-carboxamide hydrochloride, was found to be neuroprotective as it prolonged survival, ameliorated hypolocomotion, prevented weight loss, and completely prevented the atrophy of the striatal neurons (Zadori et al., 2011). These investigations suggest that KMO inhibition and/or KYNA enhancement may be neuroprotective in HD and lend support for the KP as potential drug targets.

### Multiple sclerosis

Multiple sclerosis (MS) is a chronic, demyelinating autoimmune disease of the CNS characterized by the presence of peripheral immune cells within sites of active demyelination (Carson, 2002). Based on human studies combined with experimental autoimmune encephalomyelitis (EAE), a widely-employed rodent model of MS, it is generally hypothesized that invading T-cells reactive to myelin-specific antigens are the principle effectors in MS pathogenesis (Lassmann and Ransohoff, 2004; Petermann and Korn, 2011; Fuvesi et al., 2012). Thus, a primary aim of MS research has been to define effector T-cell subpopulations relevant to disease pathogenesis and the mechanisms regulating their differentiation. As will be discussed below, accumulating evidence suggests that KP activity (1) is altered in a manner that is temporally related to the clinical course and treatment of the disease, (2) may play a role in autoimmunity by regulating T-cell differentiation, and (3) may influence the cross-talk of auto-reactive T-cells with resident microglia and infiltrating macrophages and dendritic cells.

#### Human studies implicating kynurenine pathway modulation in multiple sclerosis

Evidence for altered KP metabolism in MS first appeared in 1979 with the finding that TRP levels were significantly reduced in both plasma and CSF samples from MS patients compared with those of control subjects [Monaco et al., 1979; but see Ott et al. (1993)]. More recent studies have shown that, relative to control subjects, the downstream KP metabolite KYNA is significantly decreased in CSF of MS patients during remission, but elevated in the CSF and plasma of MS patients undergoing acute clinical exacerbation (Rejdak et al., 2002, 2007; Hartai et al., 2005). While this putative relationship between KYNA production and clinical phase has not been confirmed by single longitudinal studies, it has nevertheless collectively led to the speculation that such changes in KYNA levels during disease progression and remission reflect a compensatory protective mechanism against excitatory neurotoxicity. This hypothesis derives from the view that, as a putative NMDAR antagonist, the primary function of central KYNA is neuroprotective. However, this has not been directly tested in rodent models such as EAE as of yet. Nevertheless, these findings highlight the possibility that KP metabolism is related to the occurrence of MS and, importantly, to clinical phases of the disease.

A small number of studies have also related changes in KP metabolism to therapeutic intervention in MS patients. Therapeutically relevant concentrations of IFN-β, a standard fist-line immunomodulatory treatment for MS, leads to induction of IDO mRNA and a significant increase in the production of QUIN by human monocyte-derived macrophages (Guillemin et al., 2001). In MS patients, treatment with IFN-β leads to significant acute elevations in plasma or serum L-KYN levels and K/T ratio compared to baseline measurements, consistent with the induction of IDO in response to IFN-β (Amirkhani et al., 2005; Durastanti et al., 2011). Given the hypothesized role of KP metabolism in the mechanism underlying the depressive side-effects associated with IFN-α-based immunotherapy (Bonaccorso et al., 2002a), KP activation may be similarly involved in the depressive side-effects often reported for MS patients undergoing IFN-β treatment (Goeb et al., 2006). However, the precise relationship between IFN-β treatment and depressive symptoms in MS has not yet been definitively established, hindered in part by the partial overlap of MS symptoms with those of depression (Goeb et al., 2006). Moreover, in studies that have examined the occurrence of depressive symptoms in the context of IFN-β treatment for MS, the role that changes in KP metabolism may play has not been explored.

It has also been postulated that IFN-β-mediated IDO induction might contribute to the limited efficacy of IFN-β treatment in improving MS symptomatology (Vecsei et al., 2013). However, this idea is based on the *in vitro* finding that IFN-β leads to the production of QUIN in human monocyte-derived macrophages (Guillemin et al., 2001), incorporating the notion that QUIN is excitotoxic in the CNS (Vecsei et al., 2013). To date, though, there is no evidence that therapeutic IFN-β treatment in MS leads to central QUIN elevation as a result of IDO induction. In fact, it is not yet clear in which cell-type(s) the IFN-β-mediated IDO induction occurs in MS patients, nor which downstream KP branch is primarily affected.

#### Mechanistic insights into the role of the kynurenine pathway in multiple sclerosis: lessons from the EAE model

Since resident microglial activation and macrophage infiltration into the CNS are common features of both MS and EAE, initial interest in the role of KP metabolism in the pathogenesis of EAE arose from findings that cultured human macrophages can produce QUIN at neurotoxic levels in response to acute treatment with IFN-γ (Heyes et al., 1992; Chiarugi et al., 2001a). Indeed, in rats immunized with myelin basic protein (MBP) to induce EAE, the spinal cord concentration of QUIN is elevated compared to control rats with a time-course that closely follows the development of acute neurological symptoms, returning to control levels during remission (Flanagan et al., 1995). This presumably results from induction of IDO, but also of KMO, since anti-KMO immunoreactivity, KMO enzyme activity, as well as tissue levels of 3-HK and QUIN are enhanced in the spinal cords of EAE compared to control rats (Chiarugi et al., 2001b). Interestingly, treatment of EAE rats with the selective KMO inhibitor Ro 61-8048 significantly attenuates spinal cord 3-HK and QUIN and enhances L-KYN and KYNA, but does not alter the symptom severity in these animals (Chiarugi et al., 2001b). This observation seems to argue against a role of QUIN-mediated neurotoxicity and KYNA-mediated neuroprotection in acute clinical exacerbation and remission, respectively, in EAE and potentially MS. It does not, however, preclude a cumulative role for 3-HK and QUIN in the chronic neurodegeneration associated with secondary progressive MS.

Contrary to a contributing role in acute pathogenesis, mounting evidence from numerous EAE studies implicates IDO and specific KP metabolites in limiting autoimmunity and promoting immune tolerance, which might, in part, account for the periodic remissions observed in MS and EAE. In mice immunized with MBP or proteolipid protein 139–151 (PLP_139−151_), brain and spinal cord K/T ratio, as well as IDO mRNA and protein expression within brain and spinal cord microglia/macrophages, progressively increases with the development of EAE compared to control mice (Sakurai et al., 2002; Kwidzinski et al., 2005). However, an opposing reduction in brain and spinal cord IFN-γ mRNA during the development of EAE (Sakurai et al., 2002) suggests that IDO activity may negatively regulate the survival of IFN-γ-producing T helper type 1 (Th1) cells, thought to be a primary pathogenic T-cell subset in both MS and EAE. Consistent with this hypothesis, inhibition of IDO enzymatic activity with 1-methyl- tryptophan (1-MT) was associated with earlier relapse phase onset, significantly greater maximum clinical score, and more extensive myelitis in spinal cords of EAE mice (Sakurai et al., 2002). Similarly, EAE mice treated with 1-MT exhibit greater clinical scores during both relapse and remission phases, compared to EAE mice treated with vehicle control (Kwidzinski et al., 2005). Eliminating the possibility of off-target effects by 1-MT on exacerbation of EAE (Agaugue et al., 2006), IDO^−/−^ EAE mice exhibit more severe clinical scores compared to wildtype EAE mice, beginning approximately 2 weeks post-immunization with myelin oligodendrocyte glycoprotein (MOG)_35−55_ (Yan et al., 2010). Moreover, IDO^−/−^ mice exhibit enhanced Th1/Th17-like cytokine profiles, two major T-cell phenotypes implicated in EAE-related autoimmunity, accompanying the exacerbation of clinical symptoms in these mutants (Yan et al., 2010). Thus, a model of IDO-mediated negative feedback in EAE is emerging. IFN-γ produced by accumulating autoreactive T-cells leads to IDO induction within local antigen presenting cells (APCs), such as microglia or infiltrating macrophages and dendritic cells. This, in turn, limits the survival of pathogenic T-cell phenotypes (i.e., Th1 and Th17) and/or promotes the expansion of immunoregulatory T-cell phenotypes (i.e., Th2 and regulatory T-cells [Treg]).

A firmly established mechanism by which IDO induction may limit the survival of pathogenic T-cells is by directly reducing local availability of TRP, since it has been shown that IDO induction in macrophages and dendritic cells suppresses T-cell proliferation by local TRP catabolism (Munn et al., 1998, 1999; Mellor et al., 2003). Thus, IFN-γ-mediated IDO induction within local APCs may provide an immunosuppressive environment to control self-tolerance during inflammation. In addition to the local reduction of TRP, KP metabolites 3-hydroxykynurenic acid (3-HKA, a.k.a. xanthurenic acid), N-(3,4-dimethoxycinnamoyl) anthranilic acid (3,4-DAA), the synthetic orally active 3-HAA derivative, and 3-HAA directly suppress the proliferation of myelin-specific T-cells, specifically inhibiting Th1 and/or Th17-like phenotypes, and improving EAE clinical symptoms (Platten et al., 2005; Yan et al., 2010). At least for Th17 suppression, 3-HAA enhances the expression of TGF-β in dendritic cells (DCs), stimulating the differentiation of Tregs from naïve T-cells (Tnaïve) (Yan et al., 2010). Thus, KP metabolism may suppress autoimmunity in EAE not only through local TRP depletion, but also through the influence of KP metabolites on DC-mediated T-cell differentiation.

Though the cellular sources of the 3-HAA that act on DCs to influence T-cell differentiation is not clear, it is likely that one source of 3-HAA, or other relevant KP metabolites, may be DCs themselves since bone marrow stem cell (BMSC)-induced down-regulation of EAE correlates with IDO induction in CD11c^+^ DCs (Matysiak et al., 2008). Intriguingly, IDO induction in BMDCs and, as a consequence, Treg differentiation in BMDC/Tnaïve cocultures, requires AhR, the ligands of which include L-KYN, KYNA, and possibly other KP metabolites (Nguyen et al., 2010). In AhR^−/−^ BMDCs cocultured with Tnaïve cells, the inability of these BMDCs to induce Treg differentiation is rescued by addition of L-KYN, though it cannot be excluded that the effect of L-KYN on Treg generation is not a direct effect on Tnaïve cells (Nguyen et al., 2010) since L-KYN can also lead to AhR-dependent Treg differentiation in isolated Tnaïve cells (Mezrich et al., 2010). This may nevertheless have implications for EAE since AhR can bidirectionally drive T-cell differentiation either toward Treg or Th17 phenotypes, ameliorating or worsening EAE, respectively, depending on the specific AhR ligand (Quintana et al., 2008, 2010; Veldhoen et al., 2008). Though the effects of specific KP metabolites on AhR-mediated T-cell differentiation has not been tested directly in EAE, it is still tempting to speculate that metabolites such as 3-HAA and L-KYN might ameliorate EAE through AhR-mediated Treg differentiation, either indirectly by stimulating DC TGF-β release, or directly within Tnaïve cells.

#### Potential therapeutic intervention by modulation of kynurenine pathway in multiple sclerosis

The emerging model of KP metabolism in the underlying biology of EAE and potentially MS suggests that IDO activity, enhanced by IFN-γ released from pathogenic T-cells, may in turn serve to limit their survival and/or facilitate the expansion of immunoregulatory T-cell phenotypes during inflammation. This is postulated to occur directly through the impact of TRP catabolism on Th1/Th17 cell survival and/or by the influence of downstream KP metabolites on T-cell differentiation toward immunoregulatory phenotypes. Given the compelling positive link between IDO activity and major depressive symptoms, highlighted by clinical studies examining the depressive side-effects of IFN-α-based immunotherapy (Bonaccorso et al., 2002a), a more favorable therapeutic entry-point for MS might be based on the hypothesis that selected downstream KP metabolites serve to limit autoimmunity by influencing T-cell differentiation toward regulatory phenotypes. This hypothesis has been tested in EAE with the synthetic 3-HAA derivative N-(3,4-dimethoxycinnamoyl) anthranilic acid (3,4-DAA), also known as Tranilast, currently approved in the U.S. for the treatment of allergic rhinitis, atopic dermatitis, and certain forms of asthma (Platten et al., 2005; Chen and Guillemin, 2009; Yan et al., 2010). However, Tranilast is also proposed to inhibit histamine release by mast cells, suppress TGF-β release, and inhibit angiogenesis (Chen and Guillemin, 2009). Thus, deeper investigation into the mechanism underlying the influence of KP metabolites on T-cell differentiation may further define novel and more selective therapeutic strategies for treating autoimmune diseases such as MS in this context. To the best of our knowledge, Tranilast is currently being developed by Nuon Therapeutics, Inc. (San Mateo, CA) for the treatment of autoimmune diseases including MS, though it has not entered clinical testing.

### Epilepsy

Research efforts to investigate the function and therapeutic potential of CNS KP metabolism was originally rooted in speculation about the pro- and anti-convulsant properties of endogenous QUIN and KYNA, respectively, in the etiology of human epilepsies (Perkins and Stone, 1985; Stone and Connick, 1985; Schwarcz et al., 1987). However, in over 25 years since these ideas surfaced, surprisingly little evidence has accumulated to date, neither clinical nor experimental, to solidify alterations in KP metabolism as a major etiological factor in human epilepsy. Furthermore, the therapeutic potential of experimental KP modulators such as Ro 61-8048 and various KYNA analogs in epilepsy treatment has not been fully explored (Vecsei et al., 2013). Given this, it is not surprising that even less is known about the regulation of KP metabolism by inflammatory mediators in this context. Though outside the scope of this review, it is becoming increasingly apparent that proinflammatory cytokine signaling plays a prominent role in the mechanisms underlying neuronal hyperexcitability and neurodegeneration in epilepsy, and has been extensively reviewed elsewhere (Devinsky et al., 2013; Vezzani et al., 2013a,b). Several studies suggest that the impact of epilepsy-related neuroinflammation on KP metabolism as a disease mechanism warrants deeper investigation.

A recent study analyzed serum K/T ratios in 271 classified epilepsy patients with 309 control subjects (Liimatainen et al., 2011). Results were consistent with elevated IDO activity in patients with idiopathic generalized epilepsy (Liimatainen et al., 2011). The central KP metabolites produced downstream of IDO activation in these patients may likely be biased toward the KMO branch since microglial activation is evident in surgical resections from several forms of epilepsy (Vezzani et al., 2013a). Furthermore, in mice inoculated with hamster neurotrophic measles virus, increases in microglial activation and brain levels of 3-HK and QUIN precede the onset of behavioral seizures in this model (Lehrmann et al., 2008). Consistent with the induction of microglial IDO and KMO by proinflammatory cytokine signaling in a mouse model of temporal lobe epilepsy, hippocampal elevations in mRNA encoding IL-1β, TNF-α, IFN-γ, CD11b, IDO, and KMO were detected 24 h after kainic acid injection (Gleeson et al., 2010). Though correlative, it is plausible that these elevations in proinflammatory cytokines underlie the induction of IDO and KMO in this model since IL-1β, TNF-α, and IFN-γ are all potent inducers of IDO and at least IFN-γ also induces KMO expression as well (Mandi and Vecsei, 2012). While it may be surmised that induction of IDO and KMO likely leads to central enhancement in 3-HK and QUIN production in this model, it is not at all clear what, if any, role these metabolites might play in either acute seizure activity or in epileptogenesis. It is reasonable to hypothesize that the pro-convulsant activity of QUIN may at least exacerbate neuronal hyperactivity and/or excitotoxicity. Furthermore, both QUIN and 3-HK may contribute to neuronal degeneration to further aggravate the neuroinflammatory responses that underlie or contribute to disease pathology. To answer such questions should be relatively straightforward with the availability of molecular, genetic, and pharmacological tools to dissect the relationship between inflammatory cytokine signaling and KP metabolism in the context of epilepsy.

#### Potential therapeutic intervention by modulation of kynurenine pathway in epilepsy

While there is little clinical evidence to date supporting the notion that KP metabolism is dysregulated in epilepsy, this possibility is strengthened by our emerging understanding of the role neuroinflammation may play in the precipitation and recurrence of epileptic seizure activity, combined with the regulation of KP activity by proinflammatory cytokine signaling. Based on this and recent pre-clinical data (Lehrmann et al., 2008; Gleeson et al., 2010), we may predict that the microglial branch is overactive with respect to the astrocytic branch of the KP in at least some forms of epilepsy, resulting in excessive accumulation of 3-HK and QUIN in the CNS. If 3-HK and QUIN-mediated excitotoxicity or neurodegeneration do indeed contribute to disease pathology, then chronic, adjunctive treatment with a centrally penetrant KMO inhibitor might improve long term outcome compared to treatment with standard anti-convulsants alone, since KMO inhibition is proposed to increase the production of KYNA while decreasing the production of 3-HK and QUIN in the CNS,

### Depression and major depressive disorder

Depression is the most prevalent neuropsychological disorder. Worldwide figures estimate that ~20% of people will experience a major depressive episode throughout the course of their lifetime (Kessler et al., 2005). Understanding the etiology of major depressive disorder (MDD) is complicated by sociodemographic factors and polygenetic contributions. Emerging data show that dysregulation of the immune system, over expression of proinflammatory cytokines, and aberrant tryptophan metabolism are contributing factors at least in a subset of MDD cases.

#### Role of inflammation and kynurenine metabolism in depression from clinical and human tissue studies

Clinical evidence for an inflammation component in MDD is quite strong. The most direct argument for a causative link stems from studies in which immune stimulating agents induce depressive symptoms in patients and/or healthy subjects. A common therapy for treating hepatitis C is the use of IFN-α. Up to 50% of these patients develop depressive symptoms that are maintained throughout the course of treatment but subside within a short period after completion (Bonaccorso et al., 2002a,b). Of interest within these patients, IFN-α treatment can enhance tryptophan metabolism through the KP pathway as measured by K/T ratios, an indicator of IDO activity (Capuron et al., 2003). Tryptophan was typically reduced in serum samples, though not always (Comai et al., 2011), and kynurenine levels increased during IFN-α treatment. The alteration in K/T ratios correlated with symptoms of depression and anxiety scores on the Montgomery–Åsberg Depression Rating Scale (MADRS), Beck Depression Inventory (BDI), and Hamilton Anxiety Rating Scale (HAM-A), respectively (Bonaccorso et al., 2002b). When evaluated using the BDI scale all hepatitis C patients treated with IFN-α showed worsening scores as well as increased K/T ratios. However, only a subset (26/45) reached the criteria to be considered depressed. Interestingly, this patient subset also showed the greatest disruption in tryptophan metabolism and highest K/T ratios.

Similar to the results reported in hepatitis C populations, cancer patients treated with IFN-α also increase production of kynurenine and often possess lower tryptophan levels. In these patients K/T ratios were elevated and appeared to correlate with worsening outcome of cancer as well as development of depressive symptoms (Kurz et al., 2011). However, Bannink et al. (2007) showed that cancer patients treated with IFN-α had higher K/T ratios but did not develop symptoms of depression. It is worth noting that patients with a history of depression were excluded from this trial and as such, they may have been measuring *de novo* depression in a symptom resistant population. This interpretation would seem in line with the findings in hepatitis C patients where only a subset of patients develop depression, potentially correlating with an underlying susceptibility (Comai et al., 2011). Furthermore, the relationship between K/T ratios and depressive symptoms in melanoma patients treated with IFN-α and paroxetine supports this hypothesis. IFN-α increased K/T ratios in melanoma patients and produced depressive symptoms (Capuron et al., 2003). Paroxetine reduced the depressive symptoms and increased tryptophan, without affecting kynurenine, resulting in only modestly elevated K/T ratios. Alternatively, in patients not receiving an antidepressant, tryptophan levels were lower and kynurenine levels higher in those who developed depression compared to more resilient patients.

In healthy volunteers, stimulation of the immune system causes increased proinflammatory cytokine production and increased kynurenine production associated with depressive symptoms (Eisenberger et al., 2010). Low doses of endotoxin (from *E. coli*) increased TNF-α, IL-6, and body temperature. Interestingly, induction of the immune response correlated with reduced ventral striatum activation in a monetary incentive task, suggesting reduced function of reward systems. In other studies, higher plasma K/T ratios correlated with anhedonia scores in adolescents with MDD (Gabbay et al., 2012). Furthermore, in children with melancholic MDD, K/T, kynurenine, and 3-HAA/L-KYN levels were associated with severity of depressive symptoms (Gabbay et al., 2010).

Though a broad range of clinical studies support a role for inflammation-mediated dysregulation of cytokine production and kynurenine metabolism in MDD, some studies demonstrate a lack of correlation between inflammation, K/T ratios, and depressive symptoms. In one case, plasma IL-6 levels were reported along with a minor increase in IFN-α in a depressed cohort (Hughes et al., 2012). No evidence of increased kynurenine metabolism was observed though tryptophan was decreased. These patients also possessed elevated C-reactive protein (CRP) levels (2.1 mg/L vs. 1.2 mg/L in controls), typically used as an indicator of underlying inflammation, though they remained within a normal range. These data support the hypothesis that tryptophan depletion occurs independent of kynurenine metabolism by IDO in patients with minimal inflammation. Indeed, anti-inflammatory therapies have been found to be effective at treating depression in patients with high levels of CRP (>5 mg/L) (Raison et al., 2013). Furthermore, where it has been evaluated, proinflammatory markers such as IL-1β, TNF-α, and macrophage migration inhibitory factor appear to predict lack of responsiveness to traditional antidepressant medications (Cattaneo et al., 2013). In addition, levels of tryptophan, kynurenine, and 3-HAA correlated to treatment response to fluoxetine across a broad range of clinical scales (Mackay et al., 2009). Together these data suggest that only a subset of MDD patients with high levels of underlying inflammation are associated with disruption in kynurenine metabolism that relates to depressive symptoms.

A genetic link between inflammation and kynurenine metabolism in MDD was reported in patients with IFN-γ (+874) T/A genotypes. Healthy women with the higher IFN-γ producing T allele were associated with increased IDO activity as measured by elevated plasma levels of K/T compared to the lower producing A allele (Raitala et al., 2005). In addition, TA carriers had a higher prevalence of depression than the AA genotype (Oxenkrug et al., 2011). More recently, an IFN-γ CA repeat polymorphism was identified that also conferred lower tryptophan levels along with higher kynurenine production (Myint et al., 2013), though the relationship between symptoms of depression and kynurenine metabolism have yet to be evaluated in these patients. Furthermore, a polymorphism in the promoter region of the gene for IDO correlated with increased depression in hepatitis C patients treated with IFN-α (Smith et al., 2012). In the Sequenced Treatment Alternatives to Relieve Depression (STAR^*^D) trial two common SNPs in the IDO1 gene were associated with treatment outcome for either citalopram or overall antidepressant treatment (Cutler et al., 2012).

Though upregulated kynurenine production in serum is a relatively common finding in MDD studies, fewer reports have evaluated neuroinflammation in this disorder. QUIN is elevated in the anterior cingulate cortex of depressed patients, but only in severely depressed individuals (Steiner et al., 2011). In addition, studies have now demonstrated that, along with increased plasma kynurenine (Sublette et al., 2011), QUIN and IL-6 are increased in the cerebrospinal fluid of suicide attempters (Erhardt et al., 2013). Intriguingly, the correlation between over activation of the QUIN branch of the KP in suicide attempters was confirmed in patients with a diagnosis other than MDD as well. These data suggest that in addition to inflammation-mediated IDO activation peripherally, and perhaps within the CNS, selective metabolism of kynurenine along the QUIN branch occurs in the brains of severely depressed patients.

#### Delineation of the role of inflammation on kynurenine metabolism and depressive symptoms in preclinical systems

Preclinical studies strongly support the link between immune stimulation, induction of kynurenine metabolism, and development of depressive-like symptoms (Dantzer et al., 2011; Leonard and Maes, 2012). Acute application of an immune stimulus such as LPS induces expression of IDO, IFN-γ, TNF-α, and IL-1β in animals (O'connor et al., 2009c) while also causing impairment in forced swim (FST) and tail suspension (TST) tests, assays measuring depressive-like behavior. Blockade of IDO with 1-MT prevented the induction of IDO, attenuated increased K/T in the brain and periphery, and alleviated behavioral impairments. Interestingly IFN-γ, TNF-α, and IL-1β remained elevated suggesting that these responses to LPS occurred upstream of IDO induction. Similarly, mice treated with LPS developed an anhedonic phenotype measured by sucrose or saccharine preference which was also blocked by IDO inhibition (Salazar et al., 2012). While LPS induces sickness-like behavior which may confound the measurement of depressive-like responses in animal models, most studies demonstrate that the sickness is more transient, allowing measurement of depressive-like behavior once sickness has subsided. In fractalkine-deficient mice (CX3CR1^−/−^), chronic treatment with 1-MT prevented depressive symptoms precipitated by LPS for up to 72 h, though inhibiting IDO had no effect on sickness behavior which abated between 24 and 48 h (Corona et al., 2013).

Infusion of LPS intracerebroventricularly (icv) is used as a model of acute neuroinflammation to study the effects of cytokine regulation and depressive phenotypes in rodents. Local neuroinflammation increased kynurenine production and K/T ratios in both the CNS and in the periphery (Dobos et al., 2012). Furthermore, animals performed poorly in FST, though surprisingly no effect was observed in the elevated plus maze or in spontaneous alternation suggesting a lack of pro-anxiety responses or cognitive impairment. Inhibition of IDO with 1-MT prevented elevation of K/T as well as reduced immobility in the FST, suggesting that increased kynurenine production contributed to the depression-like phenotype. In addition to kynurenine dysregulation, icv LPS increased expression of IDO, TNF-α, IL-6, and iNOS mRNA in the brain (Fu et al., 2010). When tested acutely (4–8 h post-dose) animals also displayed significant reductions in social interaction, though it's worth noting that such an acute time period may be confounded by sickness behavior.

An alternative proinflammatory stimulus used to induce acute depressive-like responses is activation of TLR3 by Poly I:C, a synthetic dsRNA. Poly I:C induced a neuroinflammatory response characterized by transiently (<24 h) increased expression of TNF-α, IL-1β, and IL-6 with delayed increase in CD11b mRNA (24–28 h) in the frontal cortex and hippocampus of rats (Gibney et al., 2013). Depressive-like behaviors measured by saccharin preference and anxiogenic effects observed in the elevated plus maze after poly I:C treatment peaked at 48 h and persisted up to 72 h. Concurrent with the depressive phenotype, IDO expression along with tryptophan and kynurenine concentrations were elevated in the brain while no effect on 5-HT was observed. These data suggest that depressive phenotypes induced by viral-mimetic inflammation may be driven in part through dysregulation of the kynurenine system.

Chronic inflammatory stimuli also produce long-lasting depressive phenotypes associated with neuroinflammation and kynurenine dysregulation. BCG, an attenuated mycobacterium, induced an acute sickness period in mice lasting up to 5 days followed by a more prolonged depression-like phase that was sustained for weeks (Moreau et al., 2008). In this same model, kynurenine levels were increased for up to 3 weeks within the brain (Moreau et al., 2005). Dissection of the mechanism by which BCG regulates kynurenine metabolism and produces a depressive phenotype demonstrated that brain IDO, IFN-γ, and TNF-α are upregulated in concordance with depressive-like behavior. The depressive phenotype and kynurenine dysregulation produced by BCG inoculation was absent in IDO and IFN-γ KO mice (O'connor et al., 2009a,b). Furthermore, proinflammatory cytokines remained elevated in IDO KO mice, and to a lesser extent IFN-γ KO mice (e.g., IL-1β), suggesting that the impact on depression-like behaviors of cytokine induction subsequent to BCG treatment occurred through a downstream effect on kynurenine metabolism.

Both clinical and preclinical data support a link between neuroinflammation, kynurenine metabolism, and symptoms of depression. Induction of IDO, KMO, and KYNU by proinflammatory cytokines which cause disruption of normal physiological metabolism of tryptophan and/or kynurenine appears to be an important link in the cascade of events leading to certain forms of depression. Where tested in animal models, blockade of this induction has been beneficial in reversing or preventing development of depressive phenotypes. Though limited tools are available for testing the clinical benefit of manipulating the KP, it will be important for current (e.g., IDO inhibitors for the treatment of cancer) and future studies to evaluate the emotional status of patients in a systematic way to better understand the therapeutic potential of this system in MDD.

### Schizophrenia and related disorders

Schizophrenia is a complex neuropsychiatric disorder affecting approximately 1% of the world population, characterized by positive (delusions, hallucinations, thought disorder), negative (anhedonia, alogia, asociality) and cognitive (deficits in attention, executive function, and memory) symptom clusters, attributed to disturbances in dopaminergic, glutamatergic, and GABAergic neurotransmission (Harrison and Weinberger, 2005; Lewis et al., 2005). A leading hypothesis posits that NMDAR hypofunction is a key neurobiological mechanism underlying the core features of the disease, initially inspired by the observation that NMDAR open channel blockers, such as phencyclidine and ketamine, recapitulate a wide spectrum of schizophrenia symptoms in healthy subjects, and exacerbate those of schizophrenic patients [for further review, see Coyle (2012), Moghaddam and Javitt (2012)]. This, combined with the notion that KYNA is proposed to function as an endogenous antagonist of the obligatory NMDAR co-agonist site, has stimulated intense interest in the involvement of KYNA in schizophrenia. Supporting this possibility, elevated KYNA levels have been detected in CSF (Erhardt et al., 2001; Nilsson et al., 2005; Linderholm et al., 2012) and post-mortem prefrontal cortex (Schwarcz et al., 2001) of schizophrenic patients compared to controls. While human or rodent brain tissue levels of KYNA (nM range) are below the reported IC_50_ for the NMDAR co-agonist site (μM range), emerging mechanistic and behavioral data from animal studies are consistent with an impact of fluctuations in endogenous brain KYNA on schizophrenia-related phenotypes (Erhardt et al., 2009; Wonodi and Schwarcz, 2010), suggesting that local synaptic or extrasynaptic concentrations of KYNA might be much higher than the reported global levels.

#### Putative mechanisms underlying kynurenic acid dysregulation in schizophrenia and related disorders

Elevation in the level of brain KYNA may result from increased availability of L-KYN for metabolism by KAT II, the predominant KYNA-synthesizing enzyme in human and rat brain (Guidetti et al., 1997). One mechanism by which this may occur is through astrocyte-specific enhancement of L-KYN production, since brain KAT II is predominantly expressed in astrocytes compared to other neural cell types (Kiss et al., 2003; Guidetti et al., 2007b). Indeed, protein expression of TDO2 is selectively upregulated in white matter astrocytes of post-mortem frontal cortex of schizophrenic patients compared to that from control subjects, coincident with a significant elevation of TDO2 but not IDO mRNA levels (Miller et al., 2004). Similar results were obtained for post-mortem anterior cingulate cortex of subjects with schizophrenia and bipolar disorder, accompanied by an increase in tissue levels of L-KYN compared to controls (Miller et al., 2006). Thus, selective upregulation of astrocytic TDO2-mediated L-KYN synthesis may partially account for the overproduction of KYNA in brain regions implicated in cognitive impairment associated with schizophrenia. Regulatory mechanisms governing astrocytic TDO2 expression are not well-understood, though it is worth noting that the regulatory region of the gene encoding both human and rat TDO2 contain at least two glucocorticoid response elements (GREs), and TDO2 mRNA is induced by dexamethasone in rat liver (Danesch et al., 1983, 1987; Comings et al., 1995). Given this, it is tempting to speculate that, unlike the microglial branch of the KP, activity of the KYNA-producing astrocytic branch may be positively regulated by anti-inflammatory, rather than by proinflammatory signaling. This is consistent with the enhancement of brain KYNA production following administration of the COX-2 inhibitor parecoxib in rat (Schwieler et al., 2006), though the mechanism underlying this effect is unknown.

Another mechanism by which L-KYN availability for KAT II-mediated metabolism may be increased is through suppression of KMO expression and/or enzyme activity. KMO exhibits a relatively high affinity for L-KYN compared to that of KAT II, and therefore exerts preferential control over the fate of L-KYN. Thus, reduction in KMO activity is expected to increase the availability of L-KYN for KAT II-mediated metabolism, an effect which has been demonstrated experimentally using the KMO inhibitor JM-6 (Zwilling et al., 2011). Recently it has been reported that a coding SNP within the human KMO gene is associated with reduced KMO mRNA expression and elevated CSF KYNA in bipolar patients with psychotic features during mania (Lavebratt et al., 2013). Moreover, an intronic SNP within the human KMO gene is associated with reduced KMO mRNA expression and impaired schizophrenia-related endophenotypes (Wonodi et al., 2011). Thus, disease-relevant genetic impairment of KMO expression/activity might play a contributing role in the overproduction of KYNA in schizophrenia and related psychiatric disorders. It remains to be seen, however, whether KMO expression/activity may be similarly influenced by dysregulated inflammatory signaling associated with these disorders. As discussed earlier, expression of both IDO and KMO is induced by proinflammatory cytokines such as IFN-γ. Conversely, IFN-γ-mediated IDO expression is inhibited by IL-4 and IL-13 (Musso et al., 1994; Chaves et al., 2001), though opposing results have been reported (Yadav et al., 2007). Since IDO and KMO expression appear to be positively regulated by similar mechanisms, it would be interesting to determine whether KMO expression is similarly inhibited by IL-4 and/or IL-13, and whether such inhibition leads to overproduction of central KYNA. Such a hypothesis might be relevant to a role of cytokine-mediated KP dysregulation in schizophrenia, since a recent study of 26 schizophrenic patients and 26 control subjects found that the ratios of serum IFN-γ/IL-4, IFN-γ/IL-10, IL-2/IL-4, and TNF-α/IL-4 were significantly reduced in schizophrenic patients compared to controls (Chiang et al., 2013). These data support an emerging, though controversial hypothesis which proposes that schizophrenia is associated with a subtle shift from the production of Th1 cytokines, such as IFN-γ, IL-2, and TNF-α, toward the production of Th2 cytokines, such as IL-4, IL-10, and IL-6 [reviewed in Muller et al. (2012)].

#### Potential therapeutic intervention by modulation of kynurenine pathway in schizophrenia

Given the hypothesis that excessive extracellular KYNA in the CNS, through its inhibitory action at NMDAR glycineB receptors and/or α7 nicotinic acetylcholine receptors, contributes to schizophrenia symptomatology, research efforts to evaluate the therapeutic potential of reducing KYNA have focused on inhibition of KAT II, the predominant KYNA synthesizing enzyme in human and rat brain (Guidetti et al., 1997). Mounting preclinical evidence suggest that KAT II-selective inhibitors produce a pro-cognitive effect in schizophrenia-relevant behavioral assays. Acute hippocampal application of S-ESBA, a first-generation, brain-impenetrant KAT II inhibitor, reduces extracellular KYNA by ~30% and leads to improved performance in the Morris water maze, an effect that was opposed by co-administration of a physiologically relevant concentration of KYNA (Pocivavsek et al., 2011). Acute systemic administration of second-generation KAT II-selective inhibitors, such as the brain-penetrant PF-04859989 (Dounay et al., 2013) reduce central KYNA concentrations by ~70%, improve performance in a rodent sustained attention task, and reverse ketamine-induced working memory deficits in rodent and monkeys (Abbott et al., 2010; Chapin et al., 2010; Horner et al., 2010). Thus, combined with clinical and post-mortem data indicating elevated brain KYNA levels associated with schizophrenia, these pre-clinical data support the therapeutic utility of KAT II inhibitors in treating cognitive impairments associated with schizophrenia, symptom domains that are poorly treated by current standards of care.

### Attention deficit-hyperactivity disorder

Attention Deficit-Hyperactivity Disorder (ADHD) is the most commonly diagnosed psychiatric disorder in children and adolescents. Though estimates of prevalence vary widely, it is estimated that ~6–8% of school aged children suffer from this disorder (Larson et al., 2011; Willcutt, 2012). Patients show striking neuropsychological performance deficits compared to peers within their age-group which tend to diminish in severity over time. This may relate to a developmental delay of cortical maturation (Shaw and Rabin, 2009). The etiology of this delay is unknown but it has been hypothesized that impaired glial supply of energy to support neuronal activity could contribute. Recent developments in the study of ADHD suggest that patients may possess minor imbalances in their immunological systems, as measured by increased serum levels of IFN-γ and IL-13, while also having reduced levels of 3-HK though normal levels of kynurenine (Oades et al., 2010b). The altered levels of proinflammatory cytokine production and kynurenine metabolism trended toward normalizing in medicated subjects relative to medication naïve patients. These findings may be consistent with early hypotheses regarding an imbalance in tryptophan metabolism in ADHD which suggested that patients produce excess serotonin, at least in peripheral compartments (Irwin et al., 1981). An impaired production of 3-HK was predicted to reflect reduced activation of microglia and thus impaired neuronal pruning that could contribute to developmental delays.

While no study has looked directly at CNS cytokine and kynurenine profiles in ADHD, a few have attempted to define behavioral endophenotypes associated with these markers in serum. In one study it was demonstrated that levels of S100b were negatively correlated to oppositional and conduct disorder symptoms (Oades et al., 2010a). In this same study, an inverse relationship between S100b and IL-10/IL-16 was observed which was in contrast to findings in healthy children. A subsequent study reported that elevated IL-16 levels, along with reduced S100b, were strongly correlated with hyperactivity while IL-13 may be related to attentional capacity (Oades et al., 2010b). Tryptophan metabolism was not directly related to symptoms, though increased kynurenine as well as elevated IFN-γ (though reduced TNF-α) were associated with faster reaction times. Interestingly another study showed that shorter pregnancy and lower birth weight of ADHD patients, factors that are associated with severity of symptoms, have been linked to increased 3-HK and IFN-γ (Oades, 2011) which is only partially consistent with earlier reports of dysregulated cytokine production and kynurenine metabolism, where reduced 3-HK was found. While findings that alterations in peripheral cytokine and kynurenine systems are an interesting start, additional work to establish whether these results translate to changes in the CNS compartment are needed. Furthermore, a detailed analysis of cytokine levels and their relationship to kynurenine metabolism in the brain over the course of the disease may shed light on the contribution of this system to the developmental delay reported to occur in ADHD patients.

### HIV-associated neurocognitive disorder

Human Immunodeficiency Virus (HIV) infection is a debilitating chronic disease that causes dramatic CD4^+^ T-cell depletion resulting in immune response deficiency as well as chronic immune activation and inflammation responses. A strong case exists for an involvement of tryptophan metabolic disturbances in the pathology of HIV infection. Activation of tryptophan metabolism by IDO likely favors HIV persistence and exacerbation of disease progression through immune response suppression and generation of neurotoxic metabolites. Elevated circulating levels of IFN-γ and kynurenine metabolites are commonly found in HIV patients (Fuchs et al., 1990). QUIN is elevated in serum and CSF from HIV infected persons and levels are correlated with progression of neuropsychological impairment over the course of the disease (Heyes et al., 1991a). Indeed, patients with HIV-associated dementia were reported to possess levels of QUIN that are ~20-fold greater than non-infected controls. Similar increases in QUIN are observed in primate models after retroviral exposure indicating a causative link between HIV infection and activation of kynurenine metabolism (Heyes et al., 1990). However, the consequence of kynurenine dysregulation by HIV and its role in disease progression or symptomatology is unknown. Excessive activation of IDO may result in localized depletion of tryptophan availability leading to impaired T-cell differentiation, thereby suppressing immune function. In addition, inflammation-mediated induction of KMO and KYNU favors production of 3-HK and QUIN from kynurenine. 3-HK is involved in reactive oxygen species generation and also decreases the number of CD4^+^ T-cells in corneal allograph studies (Zaher et al., 2011) suggesting this neuroactive metabolite could further impair immune function after HIV infection.

The mechanism by which HIV stimulates IDO expression is not entirely clear as it has been proposed to be mediated by both IFN-γ dependent (Brown et al., 1991) and independent (Boasso et al., 2009; Maneglier et al., 2009) mechanisms in human macrophages and T-cells. To be clear, both IFN-γ levels and IDO activity are increased in HIV patients, and though IFN-γ can induce IDO, the correlation that both pathways are engaged does not necessarily indicate a causative link between these effects. Thus, while IFN-γ production, particularly from opportunistic infections, may contribute to IDO expression and tryptophan metabolism, HIV also appears to be able to stimulate kynurenine production via an interaction with CD4 receptors independent of IFN-γ. Elevated CSF kynurenine metabolism occurs independent of macrophage infiltration in simian AIDs models (Heyes et al., 1991b), suggesting that elevated QUIN is synthesized by local CNS production, possibly by microglia in response to peripheral immune/inflammation signals. Further complicating this interaction is the fact that HIV replication is enhanced by TNF-α, IFN-γ, and IL-1β, all acting through NF-κB. Since NF-κB also stimulates IDO, KMO, and KYNU, it is possible that proinflammatory cytokine signaling underlies a vicious cycle that promotes viral replication, tryptophan/kynurenine metabolism, and progression of dementia symptoms. It can thus be hypothesized that HIV infects immune cells including macrophages, T-cells, and microglia causing activation and subsequent release of proinflammatory cytokines and induction of tryptophan metabolizing enzymes. The resulting impairment in immune response could allow for opportunistic infections which further increase proinflammatory cytokine production supporting generation of 3-HK and QUIN throughout the body and brain. While the precipitating factors behind viral replication and kynurenine dysregulation may be similar, the neurocognitive dysfunction observed in HIV-associated neurocognitive disorder or dementia may be mediated in part by aberrant kynurenine metabolism in microglia within the brain in response to chronic production of proinflammatory cytokines, which one might speculate could be treated by inhibition of IDO, KMO, or KYNU.

## Therapeutic potential and immune interactions by the kynurenine pathway

The KP is uniquely positioned to regulate both the nervous and immune systems in disease states, which presents an interesting potential for drug discovery efforts but also potential risks of immunological responses. A large number of ligands targeting inhibition of kynurenine-related enzymes are available, but none have thus far advanced to clinical studies with the exception of IDO inhibitors for cancer. Decreasing production of neurotoxic metabolites such as 3-HK and QUIN with IDO, KMO, or KYNU inhibitors may reduce neuronal loss or atrophy in diseases like AD, PD, and HD, and may also offer promising therapy for MDD and HIV-associated neurocognitive disorder where neuroinflammation may contribute to the pathophysiology. Unfortunately, most ligands currently available have poor drug-like properties and would require optimization in terms of their pharmacokinetic properties. Alternatively, elevating levels of KYNA, or delivering KYNA-mimetics to the brain, may offer a different option for neuroprotection. However, it's worth noting that while KYNA analogs would be predicted to reduce excitotoxicity, it is unclear whether they could reduce oxidative stress-mediated damage caused by excessive production of 3-HK and QUIN. Furthermore, increasing KYNA may lead to psychotomimetic effects including disruption of cognitive function, whereas decreasing KYNA may be a therapeutic option for treating schizophrenia. Manipulating kynurenine metabolism for MS should be approached cautiously because the direction of change in kynurenines seems to differ depending on the stage of the disease. Also, the variability in the time course of disease progression between individuals makes it difficult to predict the stage of disease.

Evidence is emerging suggesting that IDO and downstream tryptophan catabolites play an important role in modulating immune responses. Though a review of the impact of kynurenine metabolism on the peripheral immune system is beyond the scope of the current review, it is important to keep this function of the kynurenine system in mind when considering the therapeutic potential of this pathway. An important role of IDO in immunosuppression and tolerance has been demonstrated during pregnancy, autoimmunity, resistance to tumors, and tolerance to allografts (Mellor and Munn, 2004; Barth and Raghuraman, 2012; Ban et al., 2013). Thus, therapeutic interventions involving the KP must be approached cautiously to examine the potential consequence on immunomodulatory actions. Particular attention should be paid to patients with autoimmune diseases, given that IDO may act as a negative regulator of the immune system to counteract autoimmunity. Two non-mutually exclusive theories have been proposed about how IDO and/or the kynurenine metabolites regulate the immune system: (1) IDO catabolism of tryptophan depletes this critical amino acid which is important for T-cell proliferation and (2) downstream kynurenine metabolites themselves inhibit certain immune cells (Frumento et al., 2002). Elevated IDO expression and kynurenines can have immunosuppressive effects by inhibiting CD4^+^ T-cell functions, inducing regulatory T-cells, and inhibiting Natural Killer (NK) cells (Mandi and Vecsei, 2012). Thus, the KP is directly implicated in excitatory and inhibitory neuronal communication, regulated by proinflammatory cytokines and immune signals, contributes to the production of oxidative stress, and regulates immune cell function/phenotypes, thereby positioning it to act as a key interface between the brain and immune systems. This dual role will be important to consider as the KP is mined for potential therapeutic agents to treat debilitating CNS disorders.

## Summary

Much has been learned in the thirty-some years since the first description of the KP in CNS disease. As detailed above, pathway dysregulations have been described in virtually all major CNS diseases. The pathway is sensitive to inflammatory signaling and its products have neuromodulatory properties. It has unique properties as an interface between the immune system and neuronal signaling. Inhibition of unique enzymes in this pathway has been employed to ameliorate symptomatologies in several animal models of CNS disease. Successful translation of these pre-clinical efforts into actual drugs might open the venue for novel therapeutic interventions in human CNS disease.

### Conflict of interest statement

The authors are employees of Lundbeck Research USA.
